# The Nonpenetrating Telescopic Sham Needle May Blind Patients with Different Characteristics and Experiences When Treated by Several Therapists

**DOI:** 10.1155/2011/185034

**Published:** 2011-06-20

**Authors:** Anna Enblom, Anna Johnsson, Mats Hammar, Gunnar Steineck, Sussanne Börjeson

**Affiliations:** ^1^Division of Nursing Science, Department of Medical and Health Sciences, Linköping University, 581 85 Linköping, Sweden; ^2^The Swedish Institute for Health Sciences (Vårdal Institute), Lund University, 221 00 Lund, Sweden; ^3^Department of Clinical Neuroscience, Osher Centrum, Karolinska Institute, Retzius väg 8, plan 3, 171 77 Stockholm, Sweden; ^4^Department of Oncology, Lund University Hospital, 221 85 Lund, Sweden; ^5^Department of Clinical and Experimental Medicine, Obstetrics and Gynecology, Linköping University, 581 85 Linköping, Sweden; ^6^Division of Clinical Cancer Epidemiology, Department of Oncology-Pathology, Karolinska Institute, 171 77 Stockholm, Sweden; ^7^Division of Clinical Cancer Epidemiology, Department of Oncology, Sahlgrenska Academy, 513 45 Gothenburg, Sweden; ^8^Centre of Surgery, Orthopedics and Oncology, Department of Oncology, Linköping University Hospital, Linköping, Sweden

## Abstract

*Background*. Little is known which factors influence the blinding in acupuncture studies. *Aim*. To investigate if blinding varied between patients with different characteristics receiving verum or sham acupuncture. *Methods*. We randomised cancer patients to verum (*n* = 109) or sham acupuncture (*n* = 106) with a nonpenetrating telescopic sham needle for nausea. Level of blinding was compared between different sub-groups of patients using Bang's blinding index (BI) ranged −1 to 1 (−1 = all state the opposite treatment, 1 = all identify treatment). *Results*. Most patients in the verum (74 of 95; 78%, BI 0.72) and the sham (68 of 95; 72%, BI −0.60). acupuncture group believed they had received verum acupuncture. The probability for a patient to believe he/she received verum acupuncture was related to the received needling type (*P* = .003) and to the patient's belief in received treatment effects (*P* = .008). Hospital (*P* = .425), therapist (*P* = .434), previous acupuncture experience (*P* = .578), occurrence of nausea (*P* = .157), gender (*P* = .760), and age (*P* = .357) did not affect blinding. *Conclusions*. Blinding was successfully achieved irrespective of age, gender, acupuncture experience, treatment effect, or in which hospital or by which therapist the patient received treatment. Patients with higher belief in the effect of the treatment were more likely to believe they had received verum acupuncture.

## 1. Introduction

It has been challenging for acupuncture studies to find a feasible and credible sham technique that is also as inert as possible. In randomised acupuncture studies intending to evaluate whether or not genuine (verum) acupuncture has specific effects, related to penetration of the skin and stimulation to “deqi” (a sense of soreness or numbness and a muscle twitch response), it is necessary to simulate acupuncture in the control group. Different techniques have been used: deeply inserted needles placed at nonacupuncture points, superficially inserted needles placed at acupuncture points or nonacupuncture points, ordinary but blunt needles [1, 2], or pricking with blunt devises, for example, a toothpick [3]. Penetrating sham techniques cause a greater activation in sensory areas in the midbrain compared to nonpenetrating needles [4] and increase the peripheral blood flow, irrespective of whether the needle is inserted deeply or superficially [5]. When using an ordinary but blunt acupuncture needle [6], the patient may be able to see clearly that the needle does not enter into the tissue. 

A blunt sham needle with a telescopic design needle was therefore developed by Streitberger and Kleinhenz [7] and modified by Park and coworkers [8]. When the blunt sham needle touches the surface of the skin, it gives a sensation of penetration and then glides upwards into its handle. The needle is therefore shortened, which gives the illusion that it has entered into the tissue. For evaluation of specific effects of acupuncture, consensus recommendations for acupuncture research have suggested the telescopic sham needle to be the most appropriate method of controlling for needle penetration [9]. Although telescopic nonpenetrating needles have been available for a decade, other control treatments still dominate the acupuncture research area. A review of controlled acupuncture studies in general found that 22 out of 78 reviewed studies compared acupuncture with standard care or being on a waiting list and therefore failed to distinguish between genuine and placebo effects. Forty studies performed needle insertion in the control groups, either in nontraditional acupuncture points or performed superficial insertion [10]. These are procedures previously regarded to induce significant physiological effects [4, 5]. In 16 studies within the review, the control groups received simulated acupuncture without penetration. Only a few used any of the available sham needles [10]. One item in the updated STRICTA (Standards for Reporting Interventions in Clinical Trials of Acupuncture) recommends a detailed method description of used blinding [11]. However, a review pointed out that acupuncture studies in general mostly failed to measure if blinding was successful, or in some cases, did not report the results regarding blinding [12]. Even recent reviews of acupuncture sometimes suppose that patients cannot be blinded in acupuncture efficacy studies: “Taking into account that patients and therapists are virtually impossible to be blinded to acupuncture, one point was given if the outcome assessor was blinded” [13].

Studies aiming to test the credibility of sham needles have included rather small samples of healthy individuals: *n* = 60 [7], *n* = 20 and *n* = 21 (two samples) [14], *n* = 80 [15], *n* = 20 [16], and a variety of patient samples *n* = 37 [17], *n* = 58 [18], *n* = 135 [19]. These studies suggest that individuals cannot determine if treatment has been conducted with verum or sham needles. However, there are indications that the therapist may influence the results regarding blinding [15, 17], and little is known regarding other factors potentially affecting blinding. When performing multicenter studies, it seems to be important that there are no differences in blinding results between treating centres. Under certain circumstances, a difference may produce spurious results. In addition, it is unclear whether results regarding blinding success from a small preparatory study hold when scaled fivefold. 

 We investigated if patients could identify if treatment had been given with a penetrating deqi-inducing verum acupuncture needle inserted into a traditional acupuncture point or a sham needle placed to a nonacupuncture point. Further, we studied if the level of blinding success varied between patients with different characteristics when treated by several therapists at two hospitals.

## 2. Materials and Methods

This randomised controlled study consists of two parts. The first part evaluated the effect of verum acupuncture compared to sham acupuncture regarding radiotherapy-induced nausea and vomiting, as presented previously [20]. The second part, investigating the blinding of patients, is presented here. 

### 2.1. Patients

Inclusion criteria were cancer patients of at least 18 years of age with gynaecologic, anal, rectal, colon, ventricular, pancreatic, or testicular tumours who gave their informed consent, were able to take part in the entire treatment and data collection procedure and had planned radiation over an abdominal or pelvic field of at least 800 cm^3^ volume and 25 Gray dose at one of two Swedish University Hospitals (hospital A and B). Exclusion criteria were antiemetic treatment or persistent nausea within 24 hours prior to the start of radiotherapy or acupuncture treatment during the past year for any indication or ever for nausea. 

All patients who fulfilled the study criteria from January 2004 to December 2006 consecutively received an information letter and a telephone call informing them that if they would like to participate they would receive an ordinary acupuncture treatment with needles penetrating the skin or another treatment with needles placed just against the skin.

**Figure 1:**
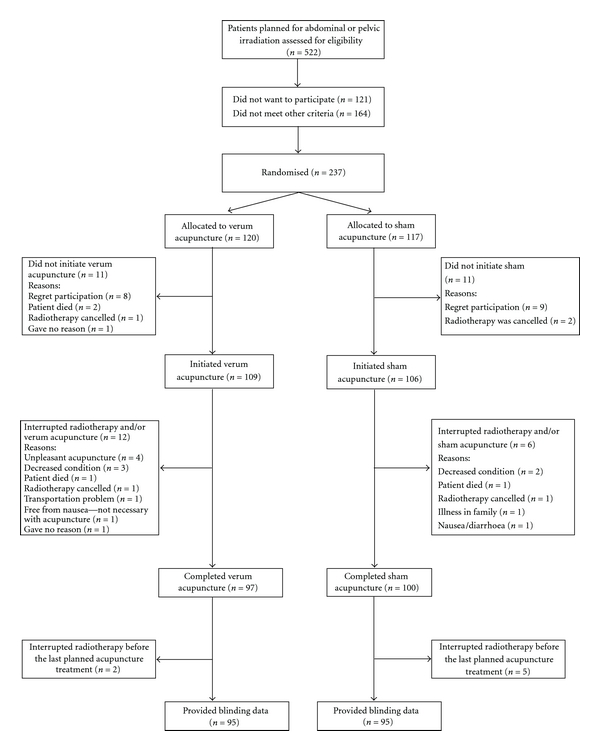
Number (*n*) of patients invited, treated and evaluated.

Patients who wanted to participate and fulfilled the study criteria were included (*n* = 237) and gave their informed consent, see Figure 1. The regional ethics committee approved the study.

### 2.2. Randomisation and Blinding

A coordinating nurse at each hospital randomised the included patients to verum or sham acupuncture—without being told which (blinded)—by drawing “lots” from an opaque envelope where allocation came from a random table. All healthcare professionals, other than the physiotherapists performing acupuncture and the coordinating nurses, were blinded. The randomising nurses were not involved in the acupuncture treatment or in obtaining patient data. 

### 2.3. Verum and Sham Acupuncture

Verum and sham acupuncture treatments started within the first day of radiotherapy and were repeated for 30 minutes three times per week during the first two weeks, followed by twice per week during the remaining individual length of the patient's radiotherapy period. Ordinary radiotherapy routines were one fraction per day Mondays to Fridays during five weeks, resulting in 12 verum or sham acupuncture treatments. Both verum and sham acupuncture needles were steel needles in sterile packaging, manufactured by Dong Bang Acupuncture (EU) LTDv. The patients were situated in a lying or sitting position during treatments.

The therapists performed verum acupuncture with sharp needles (0.30 × length 40 millimetres) bilaterally in the traditional antiemetic point pericardium six (PC6) [21] between the tendons of palmaris longus and flexor carpii radialis at two body inches (one body inch is one cun; equivalent to the greatest width of the individual patient's thumb) proximal to the wrist. The needles were inserted to a depth of a half body inch. The therapists manually stimulated the verum needles by twirling and lifting, until “deqi” occurred in the verum acupuncture group. This stimulation procedure was performed three times per treatment (every tenth minute). When the patient reported a sense of numbness or soreness and the physiotherapist noted a minimal muscular contraction around the needle (a muscle twitch response) [22], the therapists registered “deqi” in a treatment protocol. 

**Figure 2:**
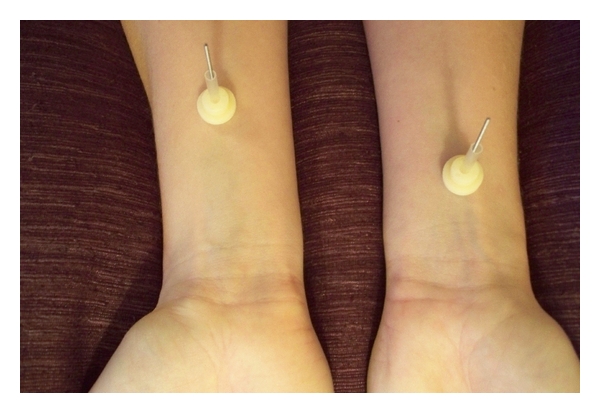
The sham needle (left) was placed at double distance from the wrist compared to the verum needle. The marking tube held the sham needles in place.

Sham acupuncture was performed with the blunt and telescopic Park's sham device [8] (0.30 × 40 millimetres when fully extended) placed bilaterally at a nonacupuncture point four cun proximal to the wrist without being inserted into the tissue (Figure 2). The therapists manually twirled and lifted the sham needles three times per treatment (every tenth minute) which resulted in the needle touching the skin but no “deqi” occurred. 

### 2.4. The Therapists at the Two Hospitals

**Figure 3:**
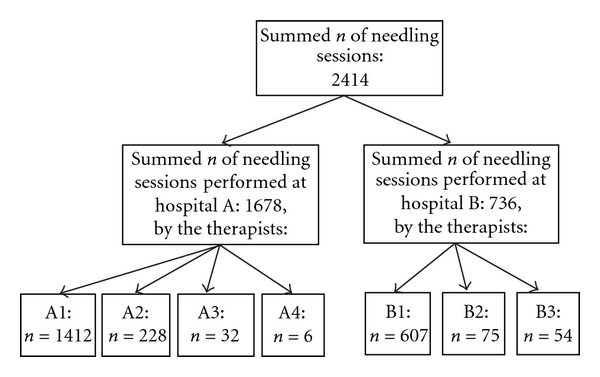
Number (*n*) of needling sessions performed at the different hospitals by the different therapists. A1 = Main therapist of hospital A, A2 = Second therapist of hospital A, and so on.

One main physiotherapist at hospital A (therapist A1) and another at hospital B (therapist B1) performed both the verum and the sham acupunctures. To ensure compliance with the planned treatment protocol, therapist A had three (therapist A2, A3, and A4) and therapist B had two (therapist B2 and B3) deputy physiotherapists. The in total seven qualified therapists performed a total number of 2414 verum and sham acupuncture treatments (Figure 3). The physiotherapists' education in acupuncture was comparable with 15 European Credit Transfer academic points (equivalent to ten weeks full-time studies). They had experience of performing acupuncture for between two and 15 years and were trained to perform sham treatments in a previous study including 80 individuals [15]. They were also trained to follow the standardised treatment protocol in a pilot study including ten patients receiving a total of 101 verum or sham acupuncture treatments [23].

### 2.5. Data Collection

#### 2.5.1. Data Regarding Patient Characteristics and Experiences of Acupuncture

The data collection procedure was before study start pilot tested [23]. At inclusion, the coordinating nurse collected medical record data (some of the collected variables are seen in Table 1). Before randomisation, the physiotherapist asked the patients “Have you previously received acupuncture?” (“Yes” or “No”). The therapists at the last treatment collected data regarding belief in received treatment effect: “Do you think that the treatment that you just received is effective in preventing and reducing nausea?” (“No, I do not think the treatment is effective”, “Yes, I believe a little”, “Moderately”, “Much” that the treatment is effective). Data regarding experience of nausea at least once during the radiotherapy period (“Yes” or “No”) were collected from the first part of the study, previously presented [20].

#### 2.5.2. Blinding Data

Bang's blinding index calculated the level of blinding success, which is a blinding index applicable irrespective of research area and has previously been used in acupuncture studies [15, 24]. The data used for calculating the index was collected immediately after the final treatment. The therapists verbally told the patients: “When initiating radiotherapy, you received an information letter and a telephone call informing you that you would receive an ordinary acupuncture treatment with needles penetrating the skin or another treatment with needles placed just against the skin” and asked “Do you think you were treated with needles that penetrated the skin, or do you think the needles were placed just against the surface of your skin?”. After answering, the patients also answered the question: “How sure are you of your answer?” using the alternatives “Not sure at all, just guessed”, “Fairly sure”, or “Entirely sure”. According to the method by Bang and coworkers [25], the participants who were not sure at all were assigned to the category: “Not sure, guessed”, whether the guess was correct or not. The patients also answered the open question: “What is the motive for your answer regarding the treatment type?” The evaluator afterwards categorised similar open answers into categories of motives. 

### 2.6. Statistics

Descriptive statistics were presented: number (*n*) and percent regarding the patients' answers in the included variables and mean and standard deviation regarding the patients' age. We compared the verum and the sham group regarding proportion of patients who believed they received penetrating acupuncture (and were entirely or moderately sure of their answer) using Fisher's exact test.

Bang's blinding index (BI) (ranged from −1 to 1) was calculated: number (*n*) of correct answers/total *n* − *n* of incorrect answers/total *n*. 1 indicates complete lack of blinding, −1 indicates opposite answers regarding treatment type, and 0 indicates perfectly conducted blinding [25]. As a first step, we compared the Bang's blinding index results and proportions of patients correctly identifying needling type in univariate analyses using Fisher's exact test and two-sided 95 percent confidence intervals between the verum and the sham acupuncture groups, and between subgroups of patients with different characteristics: patients treated at hospitals A and B, treated by therapist A1 or B1, treated by one and the same or by several therapists, in acupuncture-experienced and acupuncture-naïve patients, in men and women, in different age groups, in patients experiencing nausea or not during the radiotherapy period, and in patients with different belief in received antiemetic effects. As a second step, a multivariable logistic regression model was constructed to determine the relative importance of these studied factors (seen in Table 3–6) for explaining the probability for a patient to be fairly or entirely sure he/she had been treated with penetrating needles. The studied factors included in the analysis (Logistic procedure, forward selection) were received needling type, hospital, therapist, previous acupuncture-experience, occurrence of at least one episode of nausea within the radiotherapy-period, belief in received antiemetic effects at the last needling session, gender, and age). The significance level was set at *P* < .05.

## 3. Results

The age of the patients varied between 22 and 91 years and most patients had gynaecological tumours (Table 1). In total, 197 of the 215 randomised patients (92 percent) completed the entire verum or sham acupuncture period. None of the 18 interrupting patients did drop-out due to unblinding (Figure 1). Of the 197 completing patients, 7 provided no blinding data resulting in blinding results for 190 patients. Patient compliance is described in Figure 1. “Deqi” was registered in 1154 of 1166 performed verum acupuncture treatments and in one of 1248 performed sham treatments. In that single sham treatment, “deqi” was reached as the therapist by mistake used a verum acupuncture needle at the first stimulation.

### 3.1. Blinding Statements in the Verum and Sham Acupuncture Group

Most patients believed they had been treated with verum acupuncture, irrespective if they received verum acupuncture (74 of 95; 78%) or sham acupuncture (68 of 95; 72%) (*P* = .415). Twenty-one patients (22%) in the verum group and 84 (88%) in the sham group could not identify needling type (answered incorrect or just guessed). The Bang's blinding index result was 0.72 in the verum and −0.60 in the sham acupuncture group. According to the multivariate analysis, needling type influenced blinding (*P* = .003) (Table 2).

The patients most commonly motivated their answer regarding treatment type by that they “felt or saw signs of penetration” in both the verum (50 of 95 patients; 53%) and sham (35 of 95 patients; 37%) acupuncture group (Table 3).

### 3.2. Blinding in Patients Treated in Different Hospitals by Different Therapists

Of the 215 study patients, 145 were treated in hospital A and 70 were treated in hospital B according to the univariate analysis. In hospitals A and B, 129 (89%) and 61 (87%) of the treated patients provided blinding data. In the verum acupuncture group, there was no statistically significant difference in blinding index results between patients treated in hospital A and hospital B. In the sham acupuncture group, statistically significant more patients treated in hospital A believed they received verum acupuncture (78%, BI −0.68, 95% CI −0.83–−0.53) than in hospital B (54%, BI −0.38 (−0.67–−0.10). However, according to the multivariate analysis, the hospital where treatment was given did not influence blinding (*P* = .425) (Table 4). 

In hospital A, 96 of 145 treated patients (66%) received treatment from therapist A1 only during the entire treatment period, while 49 (34%) received treatment from two therapists (therapist A1 and *one *of the three deputy therapists A2, A3 or A4). None of the patients received treatment from more than two therapists. In hospital B, 17 (24%) of 70 treated patients were treated by therapist B1 only, 48 (69%) patients by two therapists (therapist B1 and *one* of the two deputy therapists B2 or B3), and five patients (7%) by three therapists (therapist B1 and *both* the two deputy therapists B2 and B3). Of the 96 and 17 patients treated only by therapist A1 at hospital A and therapist B1 at hospital B, 81 and 13 patients completed the whole verum/sham acupuncture period and provided blinding data, respectively. There were no statistically significant differences in blinding index results in the patients treated by therapist A1 and B1, or by patients treated by one therapist (therapist A1 or B1) or by several therapists in the verum and sham acupuncture group according to the univariate analyses. Nor according to the multivariable analysis, therapist influenced blinding (*P* = .434) (Table 4). 

### 3.3. Blinding in Patients with Different Experiences of Acupuncture

Of the 215 study patients, 210 answered the question regarding acupuncture experience and 72 of them had been treated with acupuncture previously. In the verum acupuncture group, 36 of 108 (33%) answering patients and in the sham acupuncture group 36 of 102 (35%) answering patients had been treated with acupuncture previously. Of the 96 patients treated only by therapist A1, 31 (32%) patients had previously been treated with acupuncture. Corresponding figure for the 15 answering patients (two patients did not answer the acupuncture-experience question) treated only by therapist B1 was 4 (27%).

There were no statistically significant differences in blinding index results between acupuncture-experienced and acupuncture-naïve patients according to the univariate analysis (*P* = .578) (Table 5). Blinding index results show that more of the patients that believed much they had received antiemetic effects of the treatment correctly beyond chance identified they had been treated by verum acupuncture (BI 0.71, 95% CI 0.56–0.86) compared to patients only believing little in an antiemetic effect (BI 0.60, 95% CI 0.17–1.02). The difference was statistically significant also according to the multivariate analysis (*P* = .008) (Table 5).

### 3.4. Blinding in Patients with Different Demographic Characteristics

In the verum acupuncture group, there were no sex differences in blinding results, but in the sham group, statistically significant more men (BI −0.71, 95% CI 1.02–−0.41) than women (BI −0.58, 95% CI −0.73–−0.43) believed they received verum acupuncture (Table 6). There was a tendency, however, not statistically significant according to the univariable analysis, that higher proportions of younger patients than older patients identified needling type correctly in the verum acupuncture group. According to the multivariate analysis, age did not influence blinding (*P* = .357) (Table 6). 

## 4. Discussion

We found the telescopic nonpenetrating sham needle to be a feasible sham treatment in patients receiving a series of verum or sham acupuncture; most patients believed they received verum acupuncture. Blinding was successfully achieved, in both men and women in a variety of ages, in both acupuncture-experienced and acupuncture-naïve patients with different expectations to receive antiemetic effects, irrespective of in which hospital or by which therapist the patient received treatment. Patients with higher belief in the antiemetic effect of the received treatment were more likely to believe they had received verum acupuncture, irrespective of if the patients actual experienced the treated symptom nausea or not. 

 The results regarding blinding in patients treated by verum and sham acupuncture agree with previous studies showing that individuals treated with the telescopic sham needle do not seem to recognise that a nonpenetrating treatment was given [7, 15–19]. The blinding index results of 0.72 in the verum acupuncture group and −0.61 in the sham acupuncture group in our study should be interpreted that 72% of patients treated with verum acupuncture identified beyond chance that they had received verum acupuncture, while 61% of the patients treated with sham acupuncture incorrectly believed that they had received verum acupuncture although they received the sham treatment. A positive Bang's blinding index value indicates a failure in blinding above random guessing (i.e., a majority of participants state their treatment allocation correctly), and a negative value may suggest the success of blinding or failure of blinding in the other direction (i.e., more individuals incorrectly state they were treated by the opposite treatment) [25]. In two other randomised controlled trials using the sham needle, approximately half of both verum and sham acupuncture treated patients believed they received verum acupuncture [26, 27]. If those studies had calculated Bang's blinding index, they would have received values close to 0 in both groups. A blinding index of 0 indicates that the patients randomly stated their treatment allocation: a perfectly conducted blinding of patients. However, similar to our study, more often the majority of patients believe they were treated with the “best” available option [19, 24, 28]. Kaptchuk and coworkers [28] showed that 80% of 153 patients treated with Streitberger's sham needle thought they had been treated with genuine acupuncture. This is rather similar to our study where 78% of the patients treated with sham acupuncture believed they were treated with verum acupuncture. Also in studies using placebo pills as a control, the large majority in both intervention and control groups often believe they receive the genuine treatment [29]. Bang and coworkers [25] called this trend “high response bias”, not an indication of blinding failure. When both groups believed they were treated by verum acupuncture, which was the case in our study, it is plausible that both groups had high and equally positive expectations. “High response bias” does not therefore seem to confound the measuring of the effect outcome in acupuncture studies [25, 29]. One benefit of Bang's blinding index [25] is that it presents the blinding results separately for each randomisation group and therefore has the ability to detect different behaviours in different treatment arms including the ‘response bias' example mentioned above. We did not use other available blinding index [30], for example, James' Blinding index, as it does not calculate the level of blinding in the randomisation groups separately and it does do not provide the estimate of the proportion of unblinded patients beyond random chance level. The sensorial and visual illusion of skin penetration when using the sham needle seemed to be important for the high proportion of verum and sham acupuncture treated patients believing they received verum acupuncture; they motivated their answer by the fact that they felt or saw signs of penetration or by the design or the procedure of needling. A motive-question with closed answering alternative may had increased the number of patients motivating their blinding statements, but may instead have decreased the variety in answers.

Our previous experimental study of the feasibility of the sham needle when performing one single treatment in healthy individuals indicated differences in blinding results between treating therapists [15]. In a pilot study, also White and coworkers [17] reported that blinding results varied between two therapists. In the present study, we noticed a difference between patients receiving sham acupuncture provided in hospitals A and B. However, according to the multivariate analysis the hospital where treatment was given did not influence blinding. This indicates that other factors, such as differences in belief in treatment effects, influenced blinding rather than the treating hospital itself. According to the multivariate analysis, blinding was successfully performed, irrespective of which hospital or by which therapist the patient received treatment. This tells us that when therapists have been extensively trained in a thoroughly standardised verum and sham acupuncture procedure, a rather similar and satisfactory level of blinding can be achieved. We found that previous experience of acupuncture did not seem to affect the feasibility of the sham procedure. This verifies the indication seen in a smaller study including 37 patients [17]. Many acupuncture studies [31] have chosen acupuncture experience as an exclusion criterion. Such exclusion of the large proportion of patients with acupuncture experience reduces inclusion speed or possible sample size and also decreases the possibility to generalise from these acupuncture studies. Interestingly, the subjective belief in received antiemetic effects of the treatment affected blinding; patients with higher belief in the antiemetic effect were more likely to believe they had received verum acupuncture, irrespective of if the patients actually experienced the treated symptom nausea or not.

Our rather large sample size and the fact that patients highly complied with verum or sham acupuncture and data collection and that no dropouts depended on unblinding are strengths of our study. The blinding results are based on simple verbal questions regarding believed treatment type and certainty and motives of answers. We developed these questions [15] and tested them in a pilot study (unpublished data) before this trial. A more extensive questionnaire regarding blinding was not appropriate in this frail cancer cohort, as we wanted to minimise the patient burden since the first part of this trial [20] included an ambitious data collection. It may be seen as a limitation of the study that the unblinded physiotherapists both performed treatments, asked the blinding questions and noticed the patients' answers. To minimise potential bias caused by unblinded therapists, the verum and sham acupuncture procedures were thoroughly standardised, including recommendations regarding communication and handling questions. As the therapists asked the patients the standardised blinding questions in a neutral voice, we do not believe that the unblindness of the therapists affected the blinding results but may have affected the very low report of “deqi” in the sham acupuncture group compared to other studies [7, 17, 18]. However, the muscle twitch response is not supposed to occur during sham acupuncture [22, 32]. As a more personnel consuming alternative, a blinded assistant could have asked the blinding questions. Another alternative would have been to use the sham needle developed by Takakura and Yajima [33] which may blind both patients and therapists. The therapist presses that kind of sham needle through an opaque guide tube until a stopper around the needle stops the movement. The sham needle is short enough to reach only the surface of the skin, while the verum needle is long enough to enter the tissue. Since the therapists registered “deqi”, the muscle twitch response unique to verum acupuncture treatment [22, 32], and placed the verum and sham needles in different points, blinded therapists could unfortunately not perform our study procedure. The rationale for placing the sham needles to a nonacupuncture point at the wrist was to place the needle so close that the verum and sham procedures looked rather identical, but avoid the traditional antiemetic point PC6. Verum acupuncture in PC6 selectively activated sensory areas in the mid-brain in comparison to sham acupuncture placed at a nonacupuncture point, tactile stimulation of the PC6 point, and tactile stimulation of the sham point [34]. However, we cannot expect blunt needles to be totally physiological inert; they induce a significant activation of brain areas just by touching the skin, although smaller than when using verum acupuncture needles [35]. However, the observation that the sham needle activated mid-brain sensory regions when the participants were informed that the sham was effective, while a blunt needle did not induce this activation when the individuals were told was ineffective [4], indicate the importance of a successfully performed blinding in acupuncture efficacy studies. 

To use telescopic nonpenetrating sham needles [7, 8] and place them to nonacupuncture points may be a feasible control procedure, if the researchers inquire an acupuncture look-a-like, credible control treatment that does not include important components of verum acupuncture: penetration of traditional acupuncture points, stimulated to “deqi”. The implications of this study are that after a thorough standardisation of verum and sham treatments, the nonpenetrating telescopic sham needle may, irrespective of treating therapist and treatment effect, blind both acupuncture experienced and acupuncture-naïve men and women in a variety of ages with different expectations to receive effects. Since patients with higher belief in the effect of the received treatment were more likely to believe they had received verum acupuncture, future acupuncture efficacy, studies may consider collecting data regarding patients' beliefs in the effect. 

## Figures and Tables

**Table 1:** Characteristics of the treated patients.

Characteristics	Total	Verum acupuncture	Sham acupuncture
	*n* = 215	*n* = 109	*n* = 106
*Sex, n* (%)			
Man	35 (16)	20 (18)	15 (14)
Woman	180 (84)	89 (82)	91 (86)
*Age years:* mean ± standard deviation	63.7 ± 13.8	64.3 ± 13.8	63.0 ± 13.9
*Cancer tumor type, n* (%)			
Gynecological-	147 (68)	72 (66)	75 (71)
Colon-, rectal- or anal-	60 (28)	31 (28)	29 (27)
Testicular-	2 (1)	2 (2)	0 (0)
Pancreas- or ventricular-	6 (3)	4 (4)	2 (2)

The number (*n*) of patients is presented.

**Table 2:** The ability to correctly identify if verum or sham acupuncture was given (*n* = 190).

Variable	Verum acupuncture group	Sham acupuncture group
*Certainty in statements of needling type n* (%)	*n* = 94^1^	*n* = 94^1^
Entirely sure on correct needling type	45 (48)	3 (3)
Fairly sure on correct needling type	29 (31)	8 (9)
Not sure at all, guessed correct needling type	13 (14)	6 (6)
Not sure at all, guessed opposite needling type	1 (1)	9 (10)
Fairly sure on opposite needling type	6 (6)	37 (39)
Entirely sure on opposite needling type	0 (0)	31 (33)
*Blinding answer categories according to Bang's Blinding index n* (%)	*n* = 95	*n* = 95
Stated: Needles penetrating the skin^2^	74 (78)	68 (72)
Stated: Needles placed against the skin^2^	6 (6)	11 (12)
Not sure at all, guessed	15 (16)^4^	16 (17)^5^
*Bang's blinding index^3^*	0.72 (0.60–0.83)	−0.60 (−0.74–−0.46)

The number (*n*) of patients answering the blinding question is presented. Total *n* = 190 of the entering 215 patients as 18 interrupted radiotherapy and/or verum/sham acupuncture and 7 did not provide data. ^1^ *n* = 94 since 1 patient stated he/she could not answer. ^2^The individuals were fairly or entirely sure of their answer. **^3^**Correct answers/total *n*—incorrect answers/total *n*. ^4^13 guessed correct, 1 incorrect, and 1 stated he/she could not answer. ^5^6 guessed correct, 9 incorrect, and 1 stated he/she could not answer.

**Table 3:** Patients' motives of their answers regarding blinding.

Motives of blinding statements, *n*	Treated with verum acupuncture			Treated with sham acupuncture		
	*n* = 95			*n* = 95		
	Stated: Penetrating the skin^1^	Stated: Placed against the skin^1^	Not sure at all, guessed	Stated: Penetrating the skin^1^	Stated: Placed against the skin^1^	Not sure at all, guessed
Felt or saw signs of penetration	44	2	4	29	0	6
Lack of feelings or signs of penetration	0	3	1	0	8	3
Felt effects or side-effects	2	0	0	1	0	0
Lack of effects or side-effects	0	0	0	0	0	0
Due to the design or the procedure of needling	1	0	0	3	0	0
Gave no motive^2^	27	1	10	35	3	7

*n* (number) of patients is presented. **^1^**The individuals were fairly or entirely sure of their answer. ^2^Of these 37 patients in the verum and 45 patients in the sham acupuncture group, 7 in the verum and 13 in the sham acupuncture group gave no motive at all, while 30 in the verum and 32 in the sham acupuncture group answered “It just felt like that…” (“…the needle penetrated the skin”/“…the needle was placed against the skin”).

**Table 4:** The ability to identify if verum or sham acupuncture was given in patients treated by several therapists in two hospitals.

Subgroup		Stated: Penetrating the skin^1^	Stated: Placed against the skin^1^	Not sure at all, guessed	Bang's blinding index^2^ (95% Confidence interval)	*P* value multivariate analysis^3^
*Blinding, by hospital*	*n* = 190					.425

Verum acupuncture *n* (%)						
at hospital A	*n* = 60	49 (82)	3 (5)	8 (13)	0.77 (0.63–0.90)	
at hospital B	*n* = 35	25 (71)	3 (9)	7 (20)	0.63 (0.42–0.84)	
Sham acupuncture *n* (%)						
at hospital A	*n* = 69	54 (78)	7 (10)	8 (12)	−0.68 (−0.83–−0.53)	
at hospital B	*n* = 26	14 (54)	4 (15)	8 (31)	−0.38 (−0.67–−0.10)	

*Blinding, by therapists*						.434

Verum acupuncture *n* (%)						
by one therapist (A1)	*n* = 41	35 (85)	0 (0)	6 (15)	0.85 (0.75–0.96)	
by one therapist (B1)	*n* = 9	7 (88)	0 (0)	2 (12)	0.78 (0.51–1.05)	
by two therapists (A1 and A2/A3/A4)	*n* = 19	14 (74)	3 (16)	2 (11)	0.58 (0.24–0.92)	
by two therapists (B1 and B2/B3)	*n* = 22	15 (68)	2 (9)	5 (23)	0.59 (0.32–0.86)	
by three therapists (A1 and A2/A3/A4)	*n* = 0	0 (0)	0 (0)	0 (0)	—^4^	
by three therapists (B1, B2 and B3)	*n* = 4	3 (75)	1 (25)	0 (0)	—^4^	
Sham acupuncture *n* (%)						
by one therapist (A1)	*n* = 40	31 (78)	4 (10)	5 (13)	−0.68 (−0.87–−0.47)	
by one therapist (B1)	*n* = 4	2 (50)	0 (0)	2 (50)	−0.50 (−0.99–−0.01)	
by two therapists (A1 and A2/A3/A4)	*n* = 29	23 (79)	3 (10)	3 (10)	−0.69 (−0.93–−0.45)	
by two therapists (B1 and B2/A3)	*n* = 21	11 (28)	4 (11)	6 (29)	−0.33 (−0.67–−0.00)	
by three therapists (A1 and A2/A3/A4)	*n* = 0	0 (0)	0 (0)	0 (0)	—^4^	
by three therapists (B1 and B2/B3)	*n* = 1	1 (100)	0 (0)	0 (0)	—^4^	

^1^The individuals were fairly or entirely sure of their answer. **^2^**Correct answers/total *n*—incorrect answers/total *n*. The number (*n*) of patients answering the questions is presented. Total *n* = 190 of the entering 215 patients (18 interrupted radiotherapy and/or verum/sham acupuncture and 7 did not provide blinding data). ^3^Including the variables seen in Tables 4–6 and received needling type. ^4^Blinding index was not calculated due to low *n* within the subgroup (*n* < 5). Therapist A1 = main therapist at hospital A. Therapist A2, A3, A4 = deputy therapists on hospital A.Therapist B1 = main therapist at hospital B. Therapist B2, B3 = deputy therapists on hospital B.

**Table 5:** The ability to identify if verum or sham acupuncture was given in subgroups of patients with different expectations and experiences of acupuncture.

Subgroup		Stated: Penetrating the skin^1^	Stated: Placed against the skin^1^	Not sure at all, guessed	Bang's blinding index^2^ (95% Confidence interval)	*P* value multivariate analysis^3^
*Blinding, by previous acupuncture-experience*	*n* = 185^4^					.578

Verum acupuncture *n* (%)						
acupuncture-experienced	*n* = 31	23 (74)	1 (3)	7 (23)	0.71 (0.53–0.89)	
acupuncture-naïve	*n* = 63	50 (79)	5 (8)	8 (13)	0.69 (0.53–0.85)	
Sham acupuncture *n* (%)						
acupuncture-experienced	*n* = 31	25 (81)	2 (6)	4 (13)	−0.74 (−0.94–−0.54)	
acupuncture-naïve	*n* = 60	43 (72)	8 (13)	9 (15)	−0.58 (−0.76–−0.40)	

*Blinding, by occurrence of at least one episode of nausea within the radiotherapy-period*	*n* = 190					.157

Verum acupuncture *n* (%)						
Nausea at least one episode	*n* = 63	52 (83)	4 (6)	7 (11)	0.75 (0.61–0.89)	
Free from nausea	*n* = 32	22 (69)	2 (6)	8 (25)	0.63 (0.42–0.83)	
Sham acupuncture *n* (%)						
Nausea	*n* = 56	41 (73)	6 (11)	9 (16)	−0.63 (−0.80–0.45)	
Free from nausea	*n* = 39	27 (69)	5 (13)	7 (18)	−0.56 (−0.79–−0.34)	

*Blinding, by believe in received antiemetic effects *	*n* = 190					.008

Verum acupuncture *n* (%)						
Believe not	*n* = 0	0 (0)	0 (0)	0 (0)	—^5^	
Believe little	*n* = 5	3 (60)	0 (0)	2 (40)	0.60 (0.17–1.02)	
Believe moderately	*n* = 31	25 (81)	2 (6)	4 (13)	0.79 (0.59–0.99)	
Believe much	*n* = 59	46 (78)	4 (7)	9 (15)	0.71 (0.56–0.86)	
Sham acupuncture *n* (%)						
Believe not	*n* = 1	0 (0)	1 (100)	0 (0)	—^5^	
Believe little	*n* = 3	1 (33)	1 (33)	1 (33)	—^5^	
Believe moderately	*n* = 34	23 (68)	3 (9)	8 (24)	−0.59 (−0.81–−0.37)	
Believe much	*n* = 57	44 (79)	6 (11)	7 (13)	−0.64 (−0.81–−0.48)	

^1^The individuals were fairly or entirely sure of their answer. **^2^**Correct answers/total *n*—incorrect answers/total *n*. The number (*n*) of patients answering the questions is presented. Total  *n* = 190 of the entering 215 patients (18 interrupted radiotherapy and/or verum/sham acupuncture and 7 did not provide blinding data). ^3^Including the variables seen in Tables 4–6 and received needling type. ^4^Five patients did not answer the acupuncture-experience question. ^5^Blinding index was not calculated due to low *n* within the subgroup (*n* < 5).

**Table 6:** The ability to identify if verum or sham acupuncture was given in subgroups of patients with different characteristics.

Subgroup		Stated: Penetrating the skin^1^	Stated: Placed against the skin^1^	Not sure at all, guessed	Bang's blinding index^2^ (95% Confidence interval)	*P* value multivariate analysis^3^
*Blinding, by gender*	*n* = 190					.760

Verum acupuncture *n* (%)						
Man	*n* = 18	16 (89)	1 (6)	1 (6)	0.88 (0.66–1.11)	
woman	*n* = 77	58 (75)	5 (6)	14 (18)	0.69 (0.56–0.82)	
Sham acupuncture *n* (%)						
man	*n* = 14	11 (79)	1 (7)	2 (14)	−0.71 (1.02–−0.41)*	
woman	*n* = 81	57 (70)	10 (13)	14 (17)	−0.58 (−0.73–−0.43)	

*Blinding, by age*	*n* = 190					.357

Verum acupuncture *n* (%)						
19–40 years old	*n* = 9	8 (89)	0 (0)	1 (11)	0.89 (0.68–1.09)	
41–65 years old	*n* = 40	33 (83)	2 (5)	5 (13)	0.78 (0.61–0.94)	
66–91 years old	*n* = 46	33 (72)	4 (8)	9 (18)	0.63 (0.44–0.81)	
Sham acupuncture *n* (%)						
19–40 years old	*n* = 11	6 (55)	2 (18)	3 (27)	−0.36 (−0.82–0.09)	
41–65 years old	*n* = 38	28 (74)	4 (11)	6 (16)	−0.63 (−0.84–−0.42)	
66–91 years old	*n* = 46	34 (74)	5 (11)	7 (15)	−0.63 (−0.82–−0.44)	

^1^The individuals were fairly or entirely sure of their answer. **^2^**Correct answers/total *n*—incorrect answers/total *n*. The number (*n*) of patients answering the questions is presented. Total *n* = 190 of the entering 215 patients (18 interrupted radiotherapy and/or verum/sham acupuncture and 7 did not provide blinding data. ^3^Including the variables seen in Tables 4–6 and received needling type. *Statistically significant difference between the subgroups.
